# Circular RNAs in monkey muscle: age-dependent changes

**DOI:** 10.18632/aging.100834

**Published:** 2015-11-04

**Authors:** Kotb Abdelmohsen, Amaresh C. Panda, Supriyo De, Ioannis Grammatikakis, Jiyoung Kim, Jun Ding, Ji Heon Noh, Kyoung Mi Kim, Julie A. Mattison, Rafael de Cabo, Myriam Gorospe

**Affiliations:** ^1^ Laboratory of Genetics, National Institute on Aging Intramural Research Program, National Institutes of Health, Baltimore, Maryland 21224, USA; ^2^ Translational Gerontology Branch, National Institute on Aging Intramural Research Program, National Institutes of Health, Baltimore, Maryland 21224, USA

**Keywords:** post-transcriptional gene regulation, aging, splicing, RNA-sequencing, ribonucleoprotein complex

## Abstract

Circular RNAs (circRNAs) have been identified in numerous species, including human, mouse, nematode, and coelacanth. They are believed to function as regulators of gene expression at least in part by sponging microRNAs. Here, we describe the identification of circRNAs in monkey (*Rhesus macaque*) skeletal muscle. RNA sequencing analysis was employed to identify and annotate ∼12,000 circRNAs, including numerous circular intronic RNAs (ciRNAs), from skeletal muscle of monkeys of a range of ages. Reverse transcription followed by real-time quantitative (q)PCR analysis verified the presence of these circRNAs, including the existence of several highly abundant circRNAs, and the differential abundance of a subset of circRNAs as a function of age. Taken together, our study has documented systematically circRNAs expressed in skeletal muscle and has identified circRNAs differentially abundant with advancing muscle age. We propose that some of these circRNAs might influence muscle function.

## INTRODUCTION

Aging involves the progressive accumulation of deleterious changes in cells, tissues, and organs leading to age-related physiologic declines and diseases [1]. Contributing to the functional losses and pathologies of aging are changes in gene expression programs, controlled transcriptionally and post-transcriptionally through numerous factors, including regulatory RNAs such as long noncoding (lnc)RNAs and microRNAs [2–5]. The growing family of regulatory RNAs includes a group of poorly characterized transcripts, circular RNAs (circRNAs). Although circRNAs were first identified using electron microscopy more than 35 years ago [6] only recently have we begun to understand their biological relevance. CircRNAs are highly abundant in some tissues and are rather stable molecules [7, 8], at least in part because these RNAs form circles and thus lack 5′ and 3′ ends. They are generated during splicing and can arise from exons (exonic circRNAs), introns (intronic circRNAs or ciRNAs), or a combination of exons and introns (exon-intron circRNAs or EIciRNAs) [7, 9–11]. One of the mechanisms through which circRNAs modulate gene expression is by functioning as ‘sponges’ of microRNAs, sequestering microRNAs and reducing their availability to suppress target mRNAs [7, 12]. According to other proposed molecular functions, circRNAs may also serve as sponges for RNA-binding proteins (RBPs), provide platforms for assembly of RBPs, and associate with mRNAs and modulate their expression post-transcriptionally [12].

Through these interactions, circRNAs can have a critical impact on cellular processes such as transcription, signaling, and proliferation, and on tissue and organ development and function leading to diseases such as neurodegeneration and cancer [13, 14]. For instance, some reports have documented that circRNAs are globally reduced in colorectal cancer compared to normal colon and that circRNA levels correlated negatively with proliferation in idiopathic pulmonary fibrosis and in ovarian cancer cells [14]. In developing human fetal neurons, circRNAs were found to increase broadly [15]. Specific examples illustrating these functions include the exon–intron circRNAs *EIciEIF3J* and *EIciPAIP2*, which were found to promote gene transcription [9], and *cir-ITCH*, which sponged three microRNAs (miR-7, miR-17, and miR-214) and thereby increased the levels of ITCH, an inhibitor of the Wnt/β-catenin signaling pathway [16]. In sporadic Alzheimer's disease (AD), the brain-specific circRNA ciRS-7, which contains sites that are capable of sponging miR-7, was found to be downregulated compared to age-matched control brains, likely by increasing the availability of miR-7 to brain cells in AD [13]. ciRS-7 was also implicated in tumorigenesis, as sequestration of miR-7 by ciRS-7 would be expected to promote the expression of the oncogenic factors EGFR and XIAP [17].

Skeletal muscle undergoes dramatic changes with aging, including loss of muscle mass, reduced strength, and impaired ability to regenerate, which can culminate in the development of sarcopenia [1, 18]. The physiology and pathology of skeletal muscle are profoundly influenced by microRNAs such as miR-1, miR-133, miR-206, miR-29, and miR-431 [19–21]. Given the relevance of microRNAs in muscle function and the emerging roles of circRNAs, we hypothesized that circRNAs may play key roles in processes such as muscle differentiation and regeneration, perhaps by regulating the functions of microRNAs or possibly other ligand molecules like noncoding RNAs, mRNAs, or RBPs. To begin to explore this possibility, we profiled circRNA changes with age in skeletal muscle from Rhesus monkeys (Rhesus macaque or *Macaca mulatta*). Our study provides a large number of previously unannotated circRNAs in Rhesus monkey and identifies changes in skeletal muscle circRNAs with advancing age, which may impact upon muscle aging and age-related muscle disorders.

## RESULTS

### Identification and annotation of circRNAs in monkey skeletal muscle

**Figure 1:**
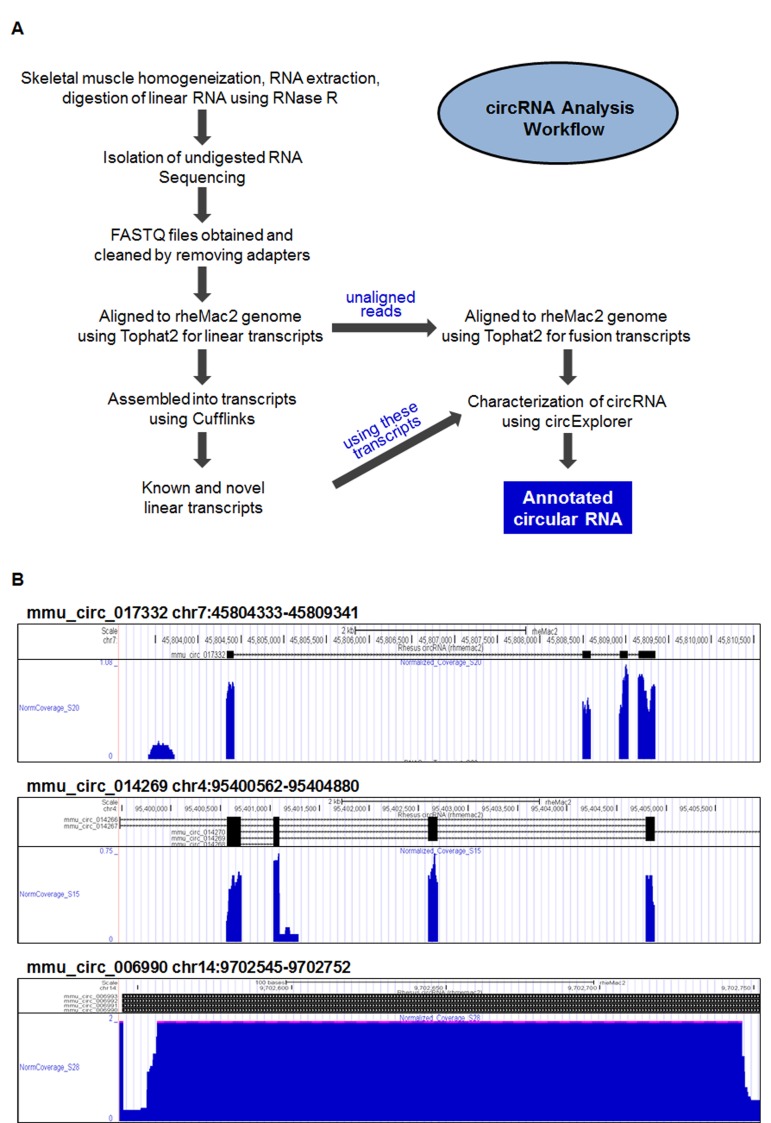
Circular RNA identification in skeletal muscle **(A)** Workflow of the preparation and analysis of circRNAs. After homogenization of skeletal muscle samples, total RNA was extracted and digested with RNase R to degrade linear RNA. Following sequencing (RNA-Seq), sequences were processed, aligned to linear transcripts, assembled into collections of known and novel circRNAs, and annotated. **(B)** Representative UCSC genome browser views of mmu_circ_017332 (*top*), mmu_circ_014269 (*middle*), and the intronic circ-RNA mmu_circ_006990 (*bottom*), including fine-scale alternate splicing patterns that generate additional circRNA isoforms.

To identify circRNAs in monkey skeletal muscle, vastus lateralis samples were obtained from monkeys with ages ranging between 0.003 and 40.9 years old. Following homogenization of the muscle tissue, total RNA was extracted, digested with RNase R, and isolated as described in the Materials and Methods section. CircRNA analysis following the steps outlined in Figure 1A identified a large number of circRNAs in monkey muscle; most of these circRNAs contained exonic sequences, but a smaller subset contained intronic sequences. For instance, circRNAs mmu_circ_017332 and mmu_circ_014269 contained exonic circRNA sequences, while mmu_circ_006990 contained intronic sequences, as illustrated in the UCSC Genome Browser view shown in Fig. 1B. A full list of the circRNAs identified is provided (Supplemental Table S1), including circRNA annotation, chromosomal coordinates, length, and accession number of the mRNA counterpart. Briefly, 12,007 circRNAs were identified as being supported by at least 2 junctional reads (2 reads from one sample or 1 read from 2 samples). Of the 9,505 circRNAs identified from sequencing all of the 24 muscle samples, 8,140 circRNAs had at least 2 reads in the initial analysis (at 20 million reads per sample, Supplemental Table S1); 1,365 circRNAs were added after in-depth sequencing (100 million reads per sample) of two additional samples [marked with an asterisk (*) in the Supplemental Table S1]. In-depth sequencing of these two additional samples led to the further addition of 2,502 circRNAs with at least two junction reads (Supplemental Table S2). The full dataset is available at GSE72879 and the circRNA database ‘circBase’.

### CircRNA abundance

We sequenced 24 muscle samples from monkeys spanning a range of ages, but initially considered all of the sequenced samples jointly. As the number of circ-RNA reads per sample varied somewhat among the 24 samples, we assessed the relative abundance of circRNAs by comparing the average of the normalized number of reads. This analysis identified about 218 circRNAs showing on average 2 or more reads among the 24 samples (each sequenced at approximately 20 million reads). The most abundant circRNAs are shown in Table 1 (full list in supplemental Table S3) and the most abundant intronic circRNAs (ciRNAs) in Table 2 (full list in supplemental Table S4).

**Table 1:** Abundant circRNAs in monkey skeletal muscle List of circRNAs most highly abundant in skeletal samples, including their respective IDs, circRNA names (genomic positions), genomic length (unspliced RNA length), and average read number in 24 samples.

Circ ID	Circ IDCircRNA name	Type	Length	Genomic length	Av. Read#
mmu_circ_010973	chr2_189036368_189048246	circRNA	247	11878	39.79
mmu_circ_016141	chr6_73699670_73701378	circRNA	181	1708	37.32
mmu_circ_019349	chr9_64242315_64243110	circRNA	172	795	32.44
mmu_circ_009828	chr18_72438948_72465599	circRNA	220	26651	30.87
mmu_circ_015464	chr6_134290742_134290950	circRNA	208	208	27.82
mmu_circ_011853	chr20_29319557_29319983	circRNA	327	426	22.98
mmu_circ_009432	chr18_14771494_14785401	circRNA	679	13907	19.57
mmu_circ_010392	chr2_102990360_103021852	circRNA	1726	31492	19.46
mmu_circ_015458	chr6_134285265_134289462	circRNA	285	4197	18.60

**Table 2:** Abundant ci (circular intronic) RNAs in monkey skeletal muscle List of the ciRNAs most highly abundant in skeletal samples, including their respective IDs, circRNA names (genomic positions), length, and average ciRNA read number in 24 samples.

Circ ID	Circ IDCircRNA name	Type	Length	Genomic length	Av. Read#
mmu_circ_013955	chr4_43067752_43067936	ciRNA	184	184	23.48
mmu_circ_007427	chr15_41837367_41837519	ciRNA	152	152	10.58
mmu_circ_001451	chr1_24464240_24464383	ciRNA	143	143	10.27
mmu_circ_001450	chr1_24464239_24464383	ciRNA	144	144	9.16
mmu_circ_007426	chr15_41837367_41837518	ciRNA	151	151	8.75
mmu_circ_006990	chr14_9702545_9702752	ciRNA	207	207	7.91
mmu_circ_004916	chr12_83327621_83328165	ciRNA	544	544	6.88
mmu_circ_008599	chr16_59500183_59500353	ciRNA	170	170	5.29
mmu_circ_006322	chr14_1750828_1750927	ciRNA	99	99	3.67

### Age-associated changes in muscle circRNAs

**Figure 2:**
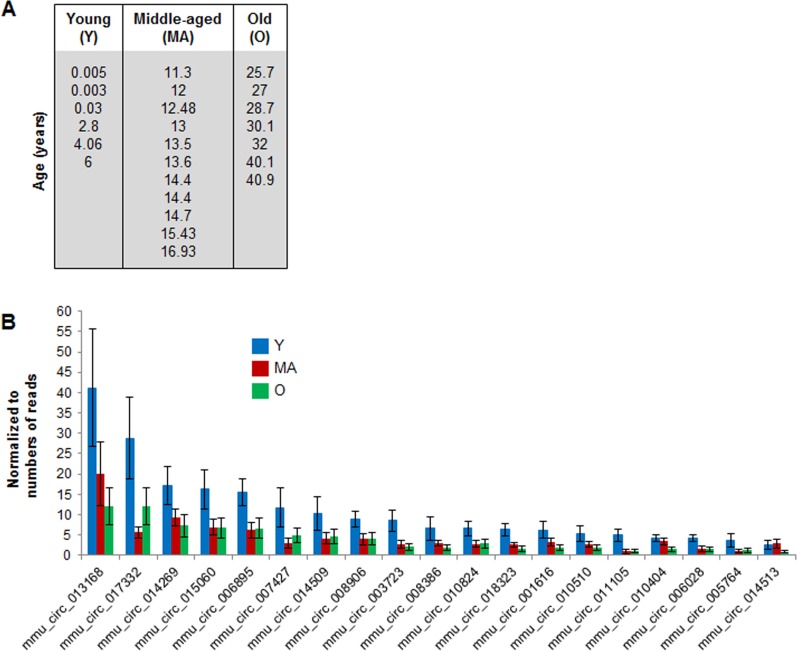
Age-associated changes in muscle circRNAs **(A)** Ages and distribution among young (Y), middle‐aged (MA) and old (O) monkeys from which skeletal muscle samples were analyzed. **(B)** Normalized read counts from circRNA‐Seq analysis showing downregulated circRNAs as a function of age.

To identify specific circRNAs displaying age-related changes in abundance, we focused on circRNAs that showed > 2 reads in at least 12 samples (Supplemental Table S5). The 218 circRNAs that met this criterion (with normalized read counts between 1 and 39) were studied further to assess possible changes in circRNA abundance with age. Six monkeys spanning 0.003 to 6 years of age were considered ‘young’ (Y), 11 monkeys 11.3- to 16.9-years old were considered ‘middle aged’ (MA), and 7 animals 25.7- to 40.9-years-old were considered ‘old’ (O) (Fig. 2A). While most circRNAs were not altered among the different age groups, some 19 circRNAs were downregulated with advancing age (Fig. 2B).

**Figure 3:**
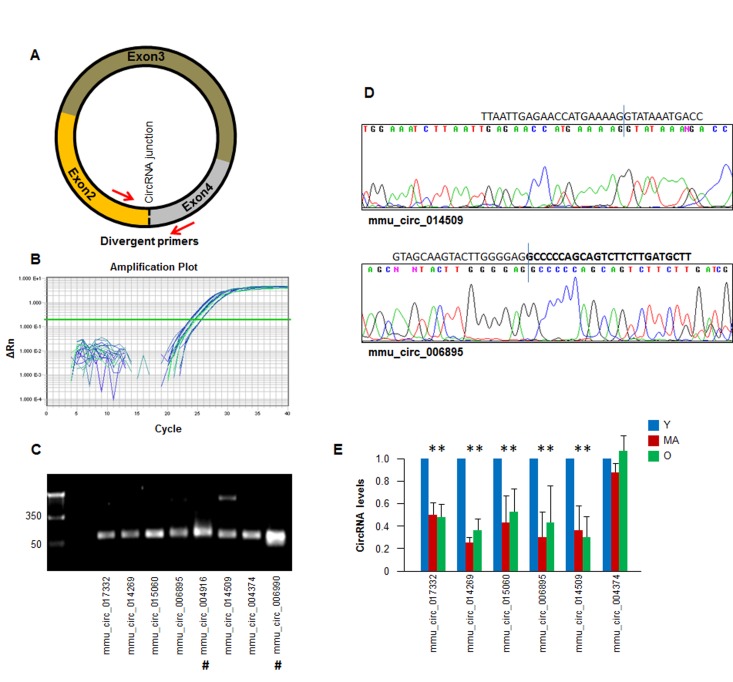
Detection of age-dependent changes in circRNA **(A)** Schematic of the design of divergent primers used for the specific detection of circRNAs. **(B)** Example of amplification plots of circRNAs in total RNA isolated monkey muscle tissues. **(C)** RT-qPCR products visualized by electrophoresis in ethidium bromide-stained agarose gels. **(D)** Representative examples of qPCR products purified and sequenced to confirm circRNA junction sequences; #, circular intronic (ci)RNAs. **(E)** For a subset of circRNAs that could be detected following the criteria outlined in the text (number of reads, pattern of dissociation curves, and single amplification products) the relative abundance in Y, MA, and O groups was quantified by RT-qPCR analysis. A circRNA that did not change with muscle age (mmu_circ_004374) was included. *, p<0.05.

To validate the results of the circRNA sequencing analysis, we designed primers in divergent orientation, specifically capable of amplifying the junction region of the circRNA (Fig. 3A; primer pairs listed in the Materials and Methods section) for the circRNA transcripts identified in Fig. 2. A subset of circRNAs detected by RT-qPCR analysis is shown (Fig. 3B). The PCR products were further assessed on agarose gels to confirm that a single DNA species was amplified in each case. Evidence of successful amplification is shown in Fig. 3C; 7 out of 8 reactions showed a single product, while mmu_circ_014509 revealed a larger band (∼350 bp), which could be a nonspecific product or another circRNA isoform. The amplified PCR products were verified by DNA sequencing to confirm the amplification of specific circRNA junctions (Fig. 3D).

Validation of the circRNAs in Fig. 2 by RT-qPCR analysis confirmed the downregulation of five circRNAs: mmu_circ_017332, mmu_circ_014269, mmu_circ_015060, mmu_circ_006895, and mmu_circ_014509; a circRNA that did not change with muscle age was included as negative control (mmu_circ_004374) (Fig. 3E). Together, these findings suggest that the abundance of most muscle circRNAs does not change with increasing age, but a specific subset of circRNAs did appear to show declining levels with age.

## DISCUSSION

Using RNA-Seq analysis, we have identified and annotated many circRNAs in Rhesus skeletal muscle. The high number (∼12,000) of circRNAs detected is in agreement with the vast number of annotated human circRNAs, which now collectively surpasses 140,000 in circBase [24]. From the large collection of muscle circRNAs, we found a subset of abundant circRNAs that displayed age-dependent changes, including a group of circRNAs showing reduced abundance with aging (Fig. 2), while virtually no circRNAs showed increased abundance with increasing age.

As these studies expand, it will be interesting to investigate the mechanisms that drive the changes in muscle circRNAs with age, particularly for the large subset of circRNAs declining in older monkeys. For example, it will be important to study whether the linear counterpart transcripts are also reduced or whether splicing machinery components giving rise to these circRNAs change with age. Similarly, assessing if these circRNAs are expressed in other tissues and if their levels in other tissues vary as a function of age warrants future scrutiny. As a class, circular RNAs appear to be well conserved among species [8]. It will be important to establish whether the human counterparts of this subset of Rhesus circRNAs are also expressed, and in particular if they are expressed in muscle, and if their expression levels change during muscle aging.

The data gathered in this report serve as a valuable platform for the identification of circRNAs that influence muscle physiology, such as muscle development, remodeling, and regeneration. To this end, work in our laboratory is underway to identify the factors (RNA molecules, DNA segments, and proteins) that interact with the circRNAs preferentially found in muscle, and in particular those that show altered levels with aging. We anticipate that some of the muscle circRNAs identified here might affect, directly or indirectly, the expression levels, localization, and/or function of myogenic transcription factors, myogenic RBPs, or myomiRs (muscle microRNAs); for instance, some of the most abundant circRNAs identified in our analysis (e.g., mmu_circ_010973, located on chromosome 2, 189036368_189048246) might be candidates for such sponging actions. It will also be interesting to investigate if these circRNAs are implicated in muscle pathologies, such as muscular dystrophy, myosarcoma, and myotonia. For abundant circRNAs showing decline with muscle aging (e.g., mmu_circ_017332 and mmu_circ_014269; Fig. 3), specific studies could be designed to assess their potential implication in sarcopenia.

In summary, we have identified collections of circRNAs abundantly expressed in skeletal muscle tissues of Rhesus monkey. Among these, we have found subsets of circRNAs showing age-associated alterations in expression. Our results constitute a valuable resource for future studies aimed at identifying circRNAs displaying tissue-, species-, and age-dependent specificity. This report contributes to the exciting emergence of circular RNAs as versatile regulators of gene expression in biological and clinical frameworks, including muscle physiology and the age-related decline in muscle function.

## MATERIALS AND METHODS

### Animals and skeletal muscle tissue

Rhesus monkeys (*Macaca mulatta*) were housed at the NIH Animal Center in Poolesville, MD. The animal center is fully accredited by the American Association for Accreditation of Laboratory Animal Care, and all of the procedures were approved by the Animal Care and Use Committee of the NIA Intramural Program. Monkeys were housed individually in standard nonhuman primate caging on a 12 h light/12 h dark cycle, at room temperature (25.5 ± 0.5°C), humidity at 60 ± 20%. Monkeys were monitored minimally 3 times daily by trained animal care staff. All monkeys were given standard chow diets (TestDiet^®^ #5038 Purina Mills, Richmond, IN or NIA-1–87, Purina Animal Nutrition LLC, Minneapolis, Minnesota) and were fed *ad libitum*. Skeletal muscle tissues (vastus lateralis) were obtained from young (Y), middle-aged (MA), and old (O) monkeys from ongoing NIA studies. Tissues were flash frozen in liquid nitrogen and stored at −80° C until assayed.

### RNA isolation, RNase R treatment, and CircRNA sequencing analysis

Tissues were homogenized in Trizol (Invitrogen, NY) and used to isolate total RNA according to the manufacturer's protocol. Total RNA (5 μg) was treated with 20 U of RNase R (RNR07250, Epicenter) for 15 min at 37°C and the digested RNA was isolated using acidic phenol-chloroform (5:1) and ethanol precipitation. CircRNA sequencing was performed at the Genomics Research Center, University of Rochester; 24 samples (one per monkey) were sequenced initially at a depth of approximately 20 million reads (paired-end), and two samples were subsequently sequenced in depth (at approximately 100 million reads) to assess sample saturation.

From the raw FASTQ files, adapter contamination was removed and sequences were aligned to the *Macaca mulatta* (rheMac2) genome with TopHat2 (v2.0.14) [22], first to identify linear RNA and later to identify fusion transcripts using reads which did not align to the linear RNA, as described [23]. RNA was assembled into linear transcripts using CuffLinks and default parameters (assembled transfrags supported by 5 fragments or more were reported) and was annotated using rheMac2 and Ensembl v78. The program circExplorer was run with the fusion transcripts obtained from TopHat2 [23] and the transcripts obtained from the assembled linear RNA (Fig. 1A).

### CircRNA sequencing, RT-qPCR analysis, DNA gel electrophoresis

Total RNA was used for reverse transcription (RT) using random hexamers and Maxima reverse transcriptase (Fermentas), and quantitative PCR (qPCR) analysis was performed using gene-specific primer pairs and SYBR Green (Kapa Biosystems). Primers to detect endogenous RNAs by RT-qPCR analysis were: CCCTATCAACTTTCGATGGTAGTCG and CCAATGGATCCTCGTTAAAGGATTT for *18S* rRNA, AATTGTCCAAGCCCTGAATG and TCTCACAGCGTCATTGGAAG for mmu_circ_017332, CCTAGAGCTGCCAAGAAGCA and TTTTCTAGTTTCATCATCAGCAGTTT for mmu_circ_014269, AAAGTGCCTGCCAAAGCTAA and TGGTTGCGTCTTTCCTTCTC for mmu_circ_015060, CAGCCAACTTTTCACCCATC and TCCTTCCAAGGAAGCTAAGTG for mmu_circ_006895, AGGCTACTCCACAGGCACAG and AAGCTCAGGTGGGTGAAGG for mmu_circ_004916, CTGCCCTCATGGACTTGAA and GGACCTTTGAACTCACCCCTA for mmu_circ_014509, GCTACATCCAGGAGAGAATGC and TTGTAGGTGTGGCTGACAAAA for mmu_circ_004374, GGCTGCATAGTGTGTGAGGA and CCAAACTGGGAAGGGAACTC for CiRNA_006990.

RT-qPCR products were size-separated by electrophoresis through ethidium bromide-stained 2% agarose gels and visualized on an ultraviolet transilluminator. Forward and reverse primers (above) were used to sequence individually the products amplified from mmu_circ_017332, mmu_circ_014269, mmu_circ_015060, mmu_circ_006895, mmu_circ_004916, mmu_circ_014509, mmu_circ_004374 and mmu_circ_006990.

## SUPPLEMENTARY MATERIAL TABLES

Supplemental Table S1 Combined circRNAs identified in skeletal muscle List of circRNAs identified from sequencing 24 samples at ∼20 million reads per sample; circRNAs were included if there were two or more instances of junction-spanning reads; in cases of 1 read, if the deeper libraries also contributed a read, those circRNAs were also included and were marked with an asterisk (*).

Supplemental Table S2 List of additional circRNAs included from the deep sequencing List of additional ciRNAs included because they showed two or more instances of junction-spanning reads from the deeper sequencing analysis (100 million reads per sample).

Supplemental Table S3 List of circRNAs used for assessment of age-dependent changes CircRNAs showing at least 2 reads in 12 or more (>50%) of the 24 muscle libraries were included in subsequent assessments of circRNAs showing differences in expression among the three age groups (Y, MA, O).

Supplemental Table S4 List of the 25 most abundant circular intronic (ci)RNAs

Supplemental Table S5 List of the 400 most abundant circRNAs
